# “Shelf” Technique Using a Novel Braided Self-Expandable Stent for the Treatment of Wide-Necked Bifurcation Aneurysms

**DOI:** 10.1007/s00062-021-01032-2

**Published:** 2021-07-20

**Authors:** Volker Maus, Werner Weber, Sebastian Fischer

**Affiliations:** Department of Diagnostic and Interventional Neuroradiology and Nuclear Medicine, Universitätsklinikum Knappschaftskrankenhaus Bochum, Universitätsklinik der Ruhr-Universität Bochum, In der Schornau 23–25, 44892 Bochum, Germany

**Keywords:** Endovascular treatment, Aneurysm treatment, Aneurysm occlusion, Stent-assisted coilembolisation, LVIS EVO

## Abstract

**Background:**

Different endovascular techniques exist for treatment of cerebral wide-necked bifurcation aneurysms (WNBA). We present the “shelf” technique with the novel woven LVIS EVO stent, which enables forming a buttress at the level of the aneurysm neck to prevent coil prolapse and additional stenting.

**Methods:**

Single-center retrospective analysis of patients treated with the “shelf” technique by using LVIS EVO stent in incidental WNBAs between January 2020 and March 2021. Inclusion criteria were saccular aneurysms with neck width ≥4 mm or a dome/neck ratio ≤2. Primary endpoint was a favorable navigation to the target vessel and successful deployment of the LVIS EVO stent with forming a buttress that enables aneurysm occlusion by subsequent coiling. Secondary endpoints were aneurysm occlusion on follow-up, procedure-related complications and clinical outcome.

**Results:**

A total of 15 patients were included. The primary end point was reached in 100% of cases. A complete aneurysm occlusion at the end of the procedure was achieved in 14/15 patients (93%). No intraprocedural complications occurred. All patients except one were discharged with an modified Rankin Scale (mRS) of 0. Procedure-related morbidity was 7%. Median follow-up imaging was 115 days (7–419 days) and available for 11/15 (73%) of the patients. Of those, 10 (91%) individuals had a complete aneurysm occlusion and 1 showed a residual neck. In all patients, the covered branch was patent and no ischemic complications occurred during follow-up.

**Conclusion:**

This study demonstrates the “shelf” technique with LVIS EVO stents as a feasible and safe treatment option for WNBAs with very good short-term occlusion rates.

## Introduction

The endovascular treatment of cerebral wide-necked bifurcation aneurysms (WNBA) is technically challenging. Neither the exclusive use of coils (with or without balloon remodeling) nor the additional use of a single stent are suitable strategies as risk of coil prolapse is high, especially for individuals with incorporation of the branch arteries into the aneurysm sac or an obtuse angle between an arising artery and the aneurysm. For this subgroup of patients, different options might be reasonable, e.g. Y‑stent assisted coiling (Y-SAC) using laser-cut stents or flow diversion. Although long-term occlusion rates of Y‑SAC are promising, it is technically complex and one of the major concerns is occurrence of ischemic complications due to the increased metal density of the overlapping stents [1]. Ischemic complications are also a main concern of extra-aneurysmal flow diverter (FD) treatment as patency of the covered side branch cannot be guaranteed after FD deployment [2]. Another treatment option might be intra-aneurysmal flow diversion, but aneurysm size, an inadequate morphology or an obtuse angle between aneurysm and parent artery can impede device implantation.

An alternative strategy for WNBAs is the “shelf” or “barrel” technique by the use of a braided stent, e.g. the low-profile visualized intraluminal support junior device (LVIS Jr., MicroVention, Aliso Viejo, CA, USA) as described previously [3, 4]. The main principle is to create a “shelf” at the entry level of the aneurysm to prevent coil prolapse and avoid the use of a second stent. Aneurysm occlusion can then subsequently be achieved by coiling through a jailed microcatheter. The novel LVIS EVO stent (MicroVention-Terumo, Aliso-Viejo, CA, USA) was recently introduced and specifically designed for SAC [5, 6]. Advantages of the stent include a proper visibility and a high metal coverage for additional flow redirection [5].

In our study, we report the feasibility, safety and short-term occlusion rate of the “shelf” technique by the use of the novel LVIS EVO stent in patients with cerebral WNBAs.

## Methods

In this single center study 27 patients non-consecutively treated with an LVIS EVO stent due to intracranial aneurysms were reviewed. The inclusion period was from January 2020 through March 2021. Baseline demographics, aneurysmal characteristics (including size, morphology and angulation grade between aneurysm and parent artery, alpha angle), technical issues, complications, and clinical outcome were noted. The institutional database was anonymized and analyzed retrospectively. Inclusion criteria were the treatment of cerebral WNBA, including anterior communicating artery (AcomA), pericallosal artery, middle cerebral artery (MCA) and internal carotid‑T, defined as neck width ≥4 mm or a dome/neck ratio ≤2, with SAC exclusively using a single LVIS EVO stent. Only saccular aneurysms were included and only elective cases were analyzed. Pretreated aneurysms and patients with preceding subarachnoid hemorrhage were also included. All indications were based on an interdisciplinary decision-making between neurosurgeons and interventional neuroradiologists.

The design of the stent has been described previously [7]. In brief, the LVIS EVO is a self-expanding and single wire-braided stent with four proximal and distal markers and has slightly flared ends. The device is available in a variety of lengths between 12 mm and 34 mm available with diameters of 2.5–4.0 mm. The stent can be recaptured up to the point of no return, which is represented by the proximal radiopaque markers. The choice of the used equipment and the applied technique for those WNBAs was left to the discretion of the operator as some of them might have been treated with other devices (e.g. intra-aneurysmal flow diversion) as well.

According to the guidelines of the respective local ethics committees, no approval was necessary for this anonymous retrospective study, which was conducted in accordance with the Declaration of Helsinki. The primary end point was technical success, defined as favorable navigation to the target vessel and successful deployment of the LVIS EVO stent with forming a buttress that enabled aneurysm occlusion by subsequent coiling with preservation of the jailed artery. The secondary end points were aneurysm occlusion rate (AOR) on follow-up imaging using the Raymond–Roy occlusion classification (RROC) [8], procedure-related complications and clinical outcome. Angiographic follow-up was exclusively assessed by digital subtraction angiography (DSA). Clinical outcome was evaluated at discharge and during follow-up according to the modified Rankin scale (mRS) score. Each patient’s angiographic and clinical status at the last follow-up was defined as the final outcome.

### Endovascular Procedure

Patients received double antiplatelet medication (75 mg/day clopidogrel, 100 mg/day aspirin) starting 5 days before the intervention and maintained for 3 months after treatment, followed by continuous single aspirin antiplatelet therapy for life if no intimal hyperplasia was detected on first angiographic follow-up. Otherwise, dual antiplatelet therapy was maintained at least for 12 months. Platelet function tests were routinely performed using ASA and P2Y12 assays (Multiplate, Roche, Basel, Switzerland).

All procedures were performed with the patient under general anesthesia by interventional neuroradiologists with at least 5 years of experience. Femoral access was obtained with a short 8F femoral sheath. Three-dimensional rotational angiography was applied in all patients to identify an ideal working projection without superimposition of surrounding vessels. All LVIS EVO stents were deployed through a dedicated 0.017″ microcatheter (Headway, Microvention, Aliso Viejo, CA, USA) using a triaxial guide-catheter system. At first, the microcatheter was navigated to a distal artery beyond the bifurcation. The choice of the branch artery was left to the operator and was dependent on vessel diameter, the bearing of the aneurysm entrance and angle between afferent and distal artery. In cases of codominant branches, the artery was used which mainly incorporated the aneurysm. Otherwise, the dominant branch artery was accessed. Then, a second microcatheter was “jailed” within the aneurysm. Subsequently, the stent was deployed with the “shelf” technique as described previously [3]. The key of the technique is to apply forward tension intermittently on the stent pusher wire and microcatheter during stent deployment after half of the aneurysm neck is covered. This leads to a further opening with expanding of the stent beyond the maximum unconstrained diameter forming a “shelf” at the entry level of the aneurysm, which prevents coil prolapse. In our experience, a stent should be chosen, which is slightly over-dimensioned to enable forming of an optimal buttress. After complete deployment of the stent, the aneurysm is occluded by the use of detachable coils.

## Results

Overall, 27 patients were treated with LVIS EVO stent during the study period; of those, 15 individuals were treated with SAC due to a cerebral WNBA. Of the patients 8 (53%) were female with a median aneurysm neck width of 3.0 mm (range 1.3–5.6 mm) and dome-to-neck ratio of 1.2 (range 0.7–1.7). Median angle between aneurysm and parent artery (alpha angle) was 26° (range 4–72°) and median age was 56 years (range 38–67 years). Of the patients two had suffered from preceding subarachnoid hemorrhage and four aneurysms were pretreated with coiling. An overview of the individual data is given in Table 1.

**Table 1:** Individual overview of patients treated with LVIS EVO “shelf” technique

No.	Age (years)/sex	Site	Dome [mm]/D/N-Ratio	Alpha angle^b^	Previous Treatment	Stent Size	Immediate RROC	Intraprocedural complication	DSA FU (days)	FU RROC	FU mRS
1	47/F	MCA	2.9/1.4	19	N	3 × 28	1	N	N/A^a^	N/A^a^	0
2	59/M	MCA	3.5/1	24	N	3 × 24	1	N	N/A^a^	N/A^a^	0
3	58/F	MCA	4.0/0.9	9	N	3 × 24	2	N	96	2	0
4	61/F	PcaA	1.8/1.2	24	Coiling	2.5 × 22	1	N	96	1	0
5	61/M	AcomA	1.3/1.0	4	N	2.5 × 22	1	N	N/A^a^	N/A^a^	0
6	56/M	MCA	1.5/1.5	26	Coiling	2.5 × 22	1	N	115	1	0
7	56/M	MCA	3.1/0.9	17	N	3 × 24	1	N	133	1	0
8	50/F	ICA‑T	3.2/1.6	47	N	3.5 × 22	1	N	193	1	0
9	38/F	AcomA	5.6/1.1	72	Coiling	2.5 × 22	1	N	89	1	0
10	52/F	MCA	1.9/1.7	29	N	2.5 × 17	1	N	98	1	0
11	65/M	AcomA	4.7/1.2	38	N	2.5 × 27	1	N	7	1	1
12	54/M	MCA	1.6/0.7	27	N	2.5 × 17	1	N	357	1	0
13	48/F	MCA	1.3/1.5	38	Coiling	3 × 24	1	N	399	1	0
14	62/F	AcomA	2.0/1.0	43	N	3 × 24	1	N	419	1	0
15	67/M	MCA	4.7/1.3	11	N	3 × 28	1	N	N/A^a^	N/A^a^	0

*AcomA* anterior communicating artery, *D/N* dome-to-neck, *DSA* digital subtraction angiography, *FU* follow-up, *MCA* middle cerebral artery, *mRS* modified Rankin scale, *N/A* not available, *PcaA* pericallosal artery, *RROC* Raymond-Roy occlusion classification, *N* no, *M* male, *F* female

^a^Still pending at the time of submission

^b^Angle between aneurysm and parent artery

**Fig. 1:**
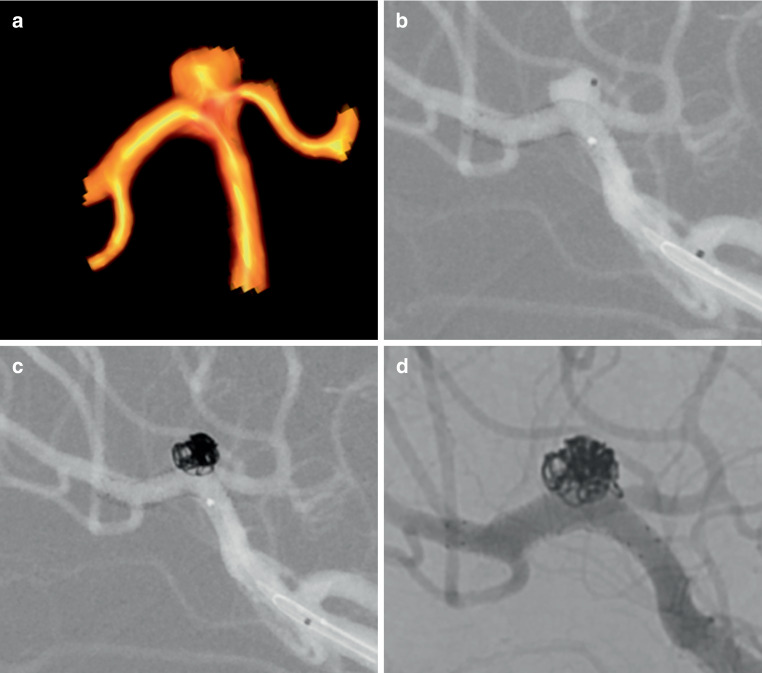
**a** Three-dimensional rotational angiography of an innocent and wide-necked bifurcation aneurysm of the middle cerebral artery in a 56-year-old-patient (patient no. 7). **b** A microcatheter was “jailed” within the aneurysm sac and a braided LVIS EVO stent (3 × 24 mm) was inserted into the dominant inferior trunk creating a shelf at the entrance of the aneurysm to prevent coil prolapse. **c** Detachment of coils through the jailed microcatheter with subsequent occlusion of the aneurysm. **d** Follow-up angiography after 133 days showed complete aneurysm occlusion, a proper contrast within the stent and patency of the covered superior trunk

**Fig. 2:**
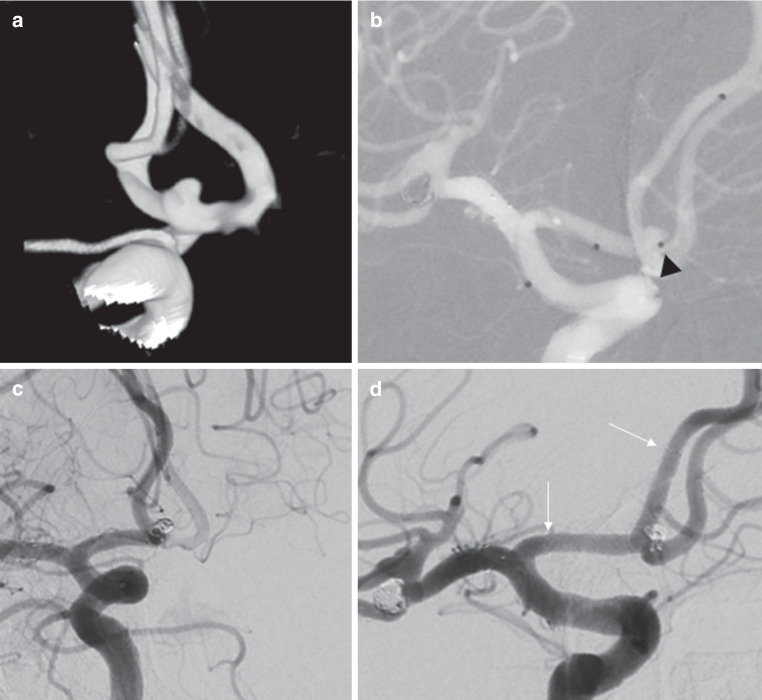
**a** Three-dimensional rotational angiography of a saccular anterior communicating artery aneurysm in a 62-year-old patient (patient no. 14). The wide-necked aneurysm was located within the bifurcation and had a dome-to-neck ratio of 1. **b** A suitable 0.017″ microcatheter was placed in “jailing” technique within the aneurysm (*black arrowhead*). Another 0.017″ microcatheter was navigated into the right A2 segment and a LVIS EVO stent (3 × 24 mm) was deployed, followed by detachment of several coils within the aneurysm. **c** On final angiogram, the aneurysm is completely occluded. **d** On follow-up angiography 419 days after treatment, the aneurysm is still occluded and the braided stent (proximal and distal end marked with *white arrow*) and the anterior communicating artery remains patent with absence of intimal hyperplasia

The primary end point was reached in 100% of cases (Figs. 1 and 2). All stents could be deployed with forming a “shelf” as intended and no coil prolapse was observed. A complete aneurysm occlusion (RROC 1) at the end of the procedure was achieved in 14/15 patients (93%) with a residual neck (RROC 2) in 1 (7%) individual. No intraprocedural complications such as stent twisting or malfunctioning of stent deployment were observed. All covered branch arteries remained patent. In 12 (80%) patients, magnetic resonance imaging was performed at the following day and 3 had silent diffusion-weighted imaging lesions. All patients except one were discharged with an mRS of 0. Procedure-related morbidity was 7% with one patient suffering from an AcomA aneurysm, who exhibited an isolated fornix infarction with an impaired retentiveness at discharge (mRS 1).

Median follow-up imaging with DSA was 115 days (range 7–419 days) and available for 11/15 (73%) patients. Of those, 10 (91%) individuals had a complete aneurysm occlusion (RROC 1) and 1 still showed an unchanged residual neck (RROC 2) at the first follow-up after 96 days. Clopidogrel was routinely discontinued but follow-up with a single antiplatelet drug was not available to date. In all patients, the covered branch was patent and no ischemic complications occurred during follow-up. One intimal hyperplasia occurred after 3 months but resolved after 1 year under maintenance of dual antiplatelet medication.

## Discussion

In this study, we present the technical feasibility, safety and short-term occlusion rate of the “shelf” technique for cerebral WNBAs using the novel LVIS EVO stent. As the intended stent deployment with subsequent coiling was feasible in all patients and the rate of complete aneurysm occlusion was high, the “shelf” technique seems to be a promising treatment option in this patient cohort.

The construction of the braided wires allows a variation in cell size property, especially if forward tension is applied during stent deployment. In a vessel bifurcation, this will lead to an expansion of the stent at the outer curve reaching a stent diameter beyond the maximum unconstrained diameter. Thus, a “shelf” is created at the level of the aneurysm entrance if force is executed after half of the aneurysm neck is covered by the stent. This technique is not advisable for laser-cut stents as open-cell stents tend to kink at the inner curve and might provoke cell entanglement [9]. Closed-cell, laser-cut stents will not reach a comparable cell size mutability within the curvature and are therefore not suitable for building an adequate buttress [3]. For the detachment of coils, it is crucial to insert the microcatheter in a “jailing” technique within the aneurysm, as navigation through the braided wires of a deployed stent is challenging and not recommended by the manufacturer. This is due to the metal coverage of the stent, which is higher compared to the LVIS and LVIS Jr. stent (28% vs. 23–27% and 17–23%, respectively) [10]. Therefore, in small aneurysms special caution is required to keep the microcatheter tip stable within the aneurysm sac during stent deployment. Another novelty of the LVIS EVO stent is the drawn filled tube technology, which makes all the wires visible under fluoroscopy and therefore facilitates stent deployment, which is helpful especially when the “shelf” is built.

**Fig. 3:**
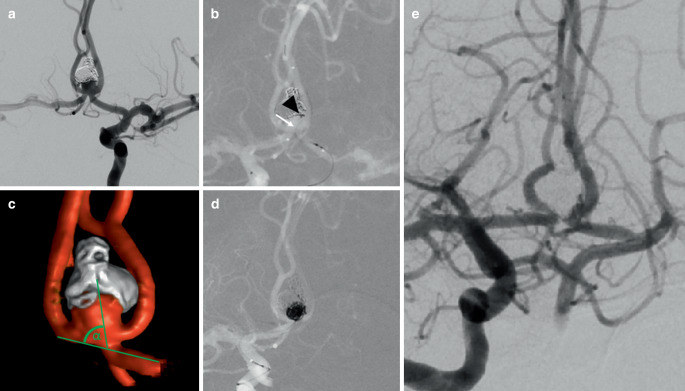
**a** Angiogram of a recurrent anterior communicating artery aneurysm in a 38-year-old patient (patient no. 9). The aneurysm was ruptured initially and treated with coiling 6 months ago. **b** After bifemoral access was obtained, the coiling microcatheter was first navigated into the aneurysm via right internal carotid artery (black arrowhead mark microcatheter tip). Then, a LVIS EVO stent (2.5 × 22 mm) was deployed through the left side (A2/A1) with forming a buttress at the level of the aneurysm neck (*white arrow*). **c** Three-dimensional rotational angiography showed a steep angle between aneurysm and parent artery (α = 72°). **d** Subsequently, the aneurysm was occluded completely by coiling. **e** Follow-up angiography revealed RROC 1 after 3 months

In comparison to other endovascular treatment strategies the “shelf” technique seems to be an alternative option in cerebral WNBAs as in our study the technical success rate was 100% with an adequate aneurysm occlusion (RROC 1 and 2) in all patients during short-term follow-up. This is comparable to a recent study of Ulfert et al., who demonstrated an adequate occlusion of 95% after 6 months by the use of pCONUS-assisted coiling [11]; however, in our study the complete occlusion rate (RROC 1) was higher with 91% in comparison to 55% in the aforementioned study. Furthermore, follow-up data were based on MRI, which delimitates evidence of in-stent intimal hyperplasia and occlusion rates. The authors mentioned a limited use of the pCONUS in patients with high angulation grades between aneurysm and parent vessel ≥70°. Even for those aneurysms, LVIS EVO “shelving” seems appropriate as in our study one patient was successfully treated with an alpha angle of 72° (Fig. 3). The barrel vascular reconstruction device was also presented as an effective device [12], but was meanwhile withdrawn from the market. The use of two stents with “Y” or “X” configuration is another effective technique with complete AOR up to 88% on long-term follow-up [13], but might have a higher complication rate due to increased metal density of the overlapping stents as described in a large study of Bartolini et al., who reported a procedure-related permanent neurologic deficits rate of 10% [14]; however, a recent meta-analysis of Y‑SAC in WNBAs also demonstrated high adequate AOR of 95% in mid-term follow-up, but a low morbidity of 2.4% [1]. Similarly, the wide-neck bifurcation aneurysms of the middle cerebral artery and the basilar apex treated by endovascular techniques (BRANCH) trial recently showed complete and adequate AOR in patients treated with coiling (including balloon-assisted and stent-assisted coiling) for unruptured WNBAs of 30.6% and 63.0%, respectively, after a follow-up of 49 weeks [15].

Another concept is intra-aneurysmal flow diversion with the Woven EndoBridge (WEB). Recently, a 3-year analysis of the European WEB Clinical Assessment of Intrasaccular Aneurysm Therapy (WEBCAST) and WEBCAST‑2 trials was published that showed adequate occlusion in 84% of cases with a low morbidity of 1.3% [16] and even our own experience with WEB is promising in unruptured, WNBAs with 78% adequate AOR at 1 year [17]. This strategy is particularly advantageous in patients with contraindications to antiplatelet therapy; however, some aneurysms might be unfavorable for WEB treatment due to very small or large aneurysm size, an inadequate morphology (e.g. daughter sac) or an obtuse angle between aneurysm and parent artery. Recently, initial results of the Contour Neurovascular System (CNS) were published but is has to be proven in future studies whether this technique might be an alternative option for WNBAs [18].

The use of extra-aneurysmal flow diversion is a matter of debate and might also be an alternative option with an adequate AOR of 79% after 1 year, but the rate of treatment-related complications is high with 21% [2]. One reason is an occlusion or at least a flow reduction of jailed arteries in 10% and 26%, respectively. In our study, no compromising of flow into jailed branches was observed.

One patient in our study suffered from fornix infarction and a mild AcomA syndrome. This might be due to occlusion of the subcallosal artery, a known AcomA perforator, which usually remains angiographically occult [19]. The perforator even in our patient was not determinable on DSA images and AcomA remained patent but distribution of the ischemic lesions suggests that the subcallosal artery was affected; however, whether this was caused by the “shelf” and the design of the LVIS EVO stent remains speculative. The procedure-related complication rate of Y‑SAC is up to 9% and is dependent on the applied stent type [1].

Long-term follow-up of aneurysms treated with the “shelf” technique is missing so far but is important with respect to definitive treatment success. As mentioned above, the high metal coverage of the braided wires provides additional flow-generating effects [5] but this will impede renavigation of a recurrent aneurysm, which might be challenging if retreatment is necessary. Renavigation might be more feasible if laser-cut or low-profile stents were primarily used; however, the rate of retreatment in SAC of cerebral WNBAs varies between 2% and 14% depending on the number of stents and stent types used [14, 20].

A limitation of our study is the retrospective design with the expected selection bias. Furthermore, the small sample size and the absence of a control group limits the validity of the data. Occlusion rates were self-assessed and results might be less favorable after core laboratory adjudication.

## Conclusion

This study demonstrates the “shelf” technique with LVIS EVO stents as a feasible and safe treatment option for WNBAs with very good short-term occlusion rates. Whether this technique may obviate the need for Y‑SAC in this patient cohort remains to be proven when long-term data are available. Therefore, further studies are necessary.
