# Correction to: “Shelf” Technique Using a Novel Braided Self-Expandable Stent for the Treatment of Wide-Necked Bifurcation Aneurysms

**DOI:** 10.1007/s00062-021-01084-4

**Published:** 2021-09-28

**Authors:** Volker Maus, Werner Weber, Sebastian Fischer

**Affiliations:** Department of Diagnostic and Interventional Neuroradiology and Nuclear Medicine, Universitätsklinikum Knappschaftskrankenhaus Bochum, Universitätsklinik der Ruhr-Universität Bochum, In der Schornau 23–25, 44892 Bochum, Germany

**Correction to:**

**Clin Neuroradiol 2021**

10.1007/s00062-021-01032-2

The original version of this article unfortunately contained a mistake. The presentation of Table 1 was incorrect. The corrected Table 1 is given below.

**Table 1:** Individual overview of patients treated with LVIS EVO “shelf” technique

No.	Age (years)/sex	Site	Dome [mm]/D/N-Ratio	Alpha angle^b^	Previous Treatment	Stent Size	Immediate RROC	Intraprocedural complication	DSA FU (days)	FU RROC	FU mRS
1	47/F	MCA	2.9/1.4	19	N	3 × 28	1	N	N/A^a^	N/A^a^	0
2	59/M	MCA	3.5/1	24	N	3 × 24	1	N	N/A^a^	N/A^a^	0
3	58/F	MCA	4.0/0.9	9	N	3 × 24	2	N	96	2	0
4	61/F	PcaA	1.8/1.2	24	Coiling	2.5 × 22	1	N	96	1	0
5	61/M	AcomA	1.3/1.0	4	N	2.5 × 22	1	N	N/A^a^	N/A^a^	0
6	56/M	MCA	1.5/1.5	26	Coiling	2.5 × 22	1	N	115	1	0
7	56/M	MCA	3.1/0.9	17	N	3 × 24	1	N	133	1	0
8	50/F	ICA‑T	3.2/1.6	47	N	3.5 × 22	1	N	193	1	0
9	38/F	AcomA	5.6/1.1	72	Coiling	2.5 × 22	1	N	89	1	0
10	52/F	MCA	1.9/1.7	29	N	2.5 × 17	1	N	98	1	0
11	65/M	AcomA	4.7/1.2	38	N	2.5 × 27	1	N	7	1	1
12	54/M	MCA	1.6/0.7	27	N	2.5 × 17	1	N	357	1	0
13	48/F	MCA	1.3/1.5	38	Coiling	3 × 24	1	N	399	1	0
14	62/F	AcomA	2.0/1.0	43	N	3 × 24	1	N	419	1	0
15	67/M	MCA	4.7/1.3	11	N	3 × 28	1	N	N/A^a^	N/A^a^	0

*AcomA* anterior communicating artery, *D/N* dome-to-neck, *DSA* digital subtraction angiography, *FU* follow-up, *MCA* middle cerebral artery, *mRS* modified Rankin scale, *N/A* not available, *PcaA* pericallosal artery, *RROC* Raymond-Roy occlusion classification, *N* no, *M* male, *F* female

^a^Still pending at the time of submission

^b^Angle between aneurysm and parent artery

The original article has been corrected.

